# New basal cell carcinoma susceptibility loci

**DOI:** 10.1038/ncomms7825

**Published:** 2015-04-09

**Authors:** Simon N. Stacey, Hannes Helgason, Sigurjon A. Gudjonsson, Gudmar Thorleifsson, Florian Zink, Asgeir Sigurdsson, Birte Kehr, Julius Gudmundsson, Patrick Sulem, Bardur Sigurgeirsson, Kristrun R. Benediktsdottir, Kristin Thorisdottir, Rafn Ragnarsson, Victoria Fuentelsaz, Cristina Corredera, Yolanda Gilaberte, Matilde Grasa, Dolores Planelles, Onofre Sanmartin, Peter Rudnai, Eugene Gurzau, Kvetoslava Koppova, Bjørn A. Nexø, Anne Tjønneland, Kim Overvad, Jon G. Jonasson, Laufey Tryggvadottir, Hrefna Johannsdottir, Anna M. Kristinsdottir, Hreinn Stefansson, Gisli Masson, Olafur T. Magnusson, Bjarni V. Halldorsson, Augustine Kong, Thorunn Rafnar, Unnur Thorsteinsdottir, Ulla Vogel, Rajiv Kumar, Eduardo Nagore, José I. Mayordomo, Daniel F. Gudbjartsson, Jon H. Olafsson, Kari Stefansson

**Affiliations:** 1 deCODE Genetics/AMGEN, Sturlugata 8, Reykjavik 101, Iceland; 2 Landspitali-University Hospital, Reykjavik 101, Iceland; 3 Faculty of Medicine, University of Iceland, Reykjavik 101, Iceland; 4 Division of Dermatology, Gregorio Marañón Hospital, Madrid 28007, Spain; 5 Division of Dermatology, Quiron Hospital, 50009 Zaragoza, Spain; 6 Division of Dermatology, San Jorge General Hospital, Huesca 22004, Spain; 7 Division of Dermatology, University Hospital, Zaragoza 50009, Spain; 8 Laboratory of Histocompatibility-Molecular Biology, Centro de Transfusión de la Comunidad Valenciana, Avenida del Cid, 65-A, Valencia 46014, Spain; 9 Department of Oncology, Instituto Valenciano de Oncologia, Valencia 46009, Spain; 10 Universidad Católica de Valencia, Valencia 46003, Spain; 11 Department of Environmental Epidemiology, National Institute of Environmental Health, Budapest H-1450, Hungary; 12 Health Department, Environmental Health Centre, Babes Bolyai University, Cluj, RO-Cluj-Napoca, Romania; 13 Department of Environmental Health, Regional Authority of Public Health, Banska Bystrica SK-975 56, Slovakia; 14 Department of Biomedicine, University of Aarhus, Aarhus C DK-8000, Denmark; 15 Danish Cancer Society Research Centre, DK-2100 Copenhagen Ø, Denmark; 16 Department of Public Health, Institute of Epidemiology and Social Medicine, University of Aarhus, Aarhus C DK-8000, Denmark; 17 Icelandic Cancer Registry, Skogarhlid 8, Reykjavik 105, Iceland; 18 Institute of Biomedical and Neural Engineering, School of Science and Engineering, Reykjavik University, Reykjavik 101, Iceland; 19 National Research Centre for the Working Environment, Copenhagen DK-2100, Denmark; 20 Division of Molecular Genetic Epidemiology, German Cancer Research Centre, Heidelberg D-69120, Germany; 21 Division of Medical Oncology, University of Colorado, Aurora, Colorado 80045, USA

## Abstract

In an ongoing screen for DNA sequence variants that confer risk of cutaneous basal cell carcinoma (BCC), we conduct a genome-wide association study (GWAS) of 24,988,228 SNPs and small indels detected through whole-genome sequencing of 2,636 Icelanders and imputed into 4,572 BCC patients and 266,358 controls. Here we show the discovery of four new BCC susceptibility loci: 2p24 *MYCN* (rs57244888[C], OR=0.76, *P*=4.7 × 10^−12^), 2q33 *CASP8-ALS2CR12* (rs13014235[C], OR=1.15, *P*=1.5 × 10^−9^), 8q21 *ZFHX4* (rs28727938[G], OR=0.70, *P*=3.5 × 10^−12^) and 10p14 *GATA3* (rs73635312[A], OR=0.74, *P*=2.4 × 10^−16^). Fine mapping reveals that two variants correlated with rs73635312[A] occur in conserved binding sites for the GATA3 transcription factor. In addition, expression microarrays and RNA-seq show that rs13014235[C] and a related SNP rs700635[C] are associated with expression of *CASP8* splice variants in which sequences from intron 8 are retained.

## Abstract (web summary)

Basal cell carcinoma is a common cancer among people of European ancestry, with associated high economic costs to monitor and treat. Here Stacey *et al.* conduct a genome-wide association study on Icelandic and other European populations, identifying four novel loci associated with cancer susceptibility.

## Graphical abstract

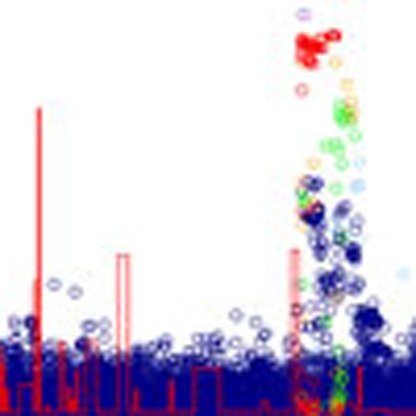

Basal cell carcinoma (BCC) is the most common cancer type in populations of European ancestry and its incidence has been increasing. Although it rarely metastasizes, it can be locally invasive and can cause considerable morbidity. Forty to fifty per cent of affected individuals develop new primary lesions within five years. The economic burden of treating and monitoring BCC is substantial^1^,^2^. In common with other forms of skin cancer, ultraviolet exposure is a major risk factor^3^.

Genetics plays a substantial role in BCC. Rare, high-penetrance inherited mutations in the Hedgehog pathway genes *PTCH1*, *PTCH2* and *SUFU* cause Gorlin Syndrome (also called basal cell nevus syndrome)^1^,^4^,^5^. Somatic mutations in Hedgehog pathway genes, in addition to *TP53*, are commonly observed in BCCs^1^,^6^. Genome-wide association and candidate gene studies have yielded a substantial number of low-to-moderate penetrance sequence variants that predispose to BCC. Some of the genes implicated, such as *MC1R*, *ASIP*, *TYR*, *SLC45A2*, *OCA2* and *IRF4* also affect pigmentation in Europeans^7^,^8^,^9^,^10^,^11^,^12^,^13^. Their effects on BCC risk are thought to be mediated at least in part by their influence on responses to ultraviolet exposure. Other genes implicated in BCC predisposition include *PADI6/RCC2*, *RHOU*, *TERT*, *KRT5*, *CDKN2A/B*, *KLF14*, *TP53*, *TGM3* and *RGS22*, which have no obvious relation to ultraviolet-sensitive pigmentation traits^10^,^14^,^15^,^16^,^17^.

The Icelandic Cancer Registry has recorded histopathologically confirmed diagnoses of BCC since 1981, making studies using population-based case ascertainment possible^18^. We are conducting a search for BCC susceptibility loci based on variants discovered through whole-genome sequencing in the Icelandic population^15^. We use long-range phasing and imputation to determine the genotypes of these variants for single-nucleotide polymorphism (SNP) microarray chip-typed individuals and their relatives^15^,^19^,^20^. We now report that we have sequenced the whole genomes of 2,636 Icelanders to a median depth of 20 × . Genotypes of the variants detected by sequencing were imputed into 4,572 individuals with BCC (cases) and 266,358 controls using an algorithm that has been improved since our previous study^16^,^21^. Association analysis and replication genotyping in 1,475 cases and 4,733 control samples from non-Icelandic populations revealed evidence for four new BCC susceptibility loci.

## Results

### Loci associated with BCC

Imputation of genotypes for the variants identified by whole-genome sequencing allowed us to perform 24,988,228 tests of association; 19,543,184 tests for SNPs and 5,445,044 tests for small indels (<60 bp), testing only alleles that could be imputed with an information value above 0.8. Association testing was performed using logistic regression, treating disease status as the response and genotype counts as covariates. A Q–Q plot is shown in Supplementary Fig. 1. In addition to reconfirming known BCC susceptibility loci, we identified four new loci that displayed substantial association signals (Supplementary Fig. 2).

**Figure 1:**
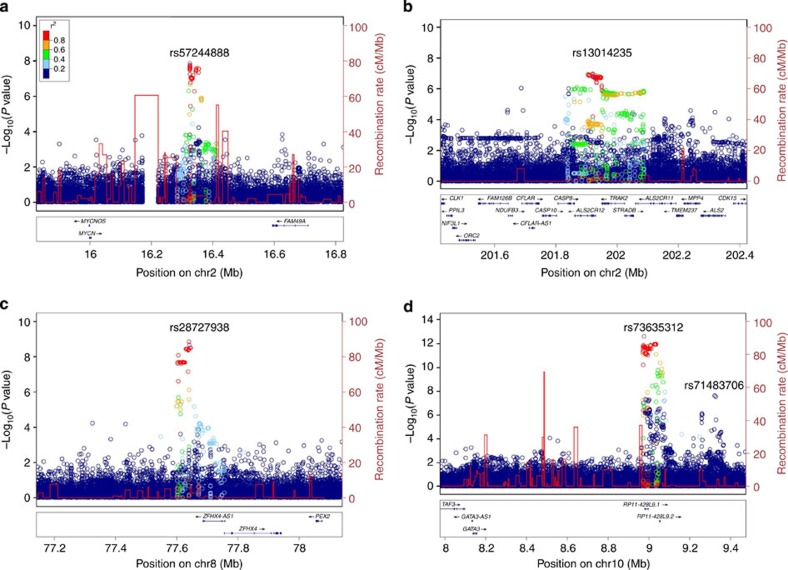
New BCC association signals. Loci are (**a**) 2p24 *MYCN*, (**b**) 2q33 *CASP8-ALS2CR12*, (**c**) 8q21 *ZFHX4* and (**d**) 10p14 *GATA3* loci in the Icelandic sample. Data are based on association signals (expressed as −log_10_(*P*) derived by logistic regression) for variants identified by whole-genome sequencing and imputation. The index SNP at each locus is labelled. Other variants are plotted in colours according to their *r*^2^ values relative to the index SNP as indicated in the legend (**a**). Recombination rates, in cM/Mb and based on Icelandic data, are plotted as red lines. The lower panel sections shows the locations of RefSeq genes and the chromosomal position (hg18 Build 36). The regions shown are ±500 kb from the index SNP except for 10p14 *GATA3*, where the region is extended to −1 Mb to show the location of the *GATA3* gene.

We observed BCC association signals at 2p24 from a group of variants with minor allele frequencies around 10.2%. The strongest signal came from rs57244888[C], with odds ratio (OR)=0.77, *P*=1.4 × 10^−8^ (Fig. 1a, Table 1). The peak is in the intergenic region between *MYCN* and *FAM49A* and is separated from both genes by regions of moderate recombination rates (Fig. 1a). We generated a single-track Centaurus assay for rs57244888 and genotyped it in replication samples from Spain, Denmark and eastern Europe. The association replicated significantly in the non-Icelandic samples (OR=0.74, *P*=6.9 × 10^−5^) and there was no evidence of heterogeneity between the sample sets (Table 1). Combining the Icelandic and non-Icelandic data provided strong overall evidence of an association (OR=0.76, *P*=4.7 × 10^−12^).

At 2q33, there were signals from a group of variants with population minor allele frequencies of about 46%, represented by rs13014235[C] (OR=1.15, *P*=1.6 × 10^−7^, Fig. 1b). Significant replication was obtained in non-Icelandic sample sets (OR=1.15, *P*=0.0027) and combining all data sets yielded an OR=1.15, *P*=1.5 × 10^−9^ (Table 1). rs13014235 is a p.Val34Leu missense variant in *ALS2CR12* (amyotrophic lateral sclerosis 2 chromosomal region, candidate gene 12). The *ALS2CR12* gene product is a structural component of the sperm flagellum^22^. Considered together with SIFT and PolyPhen predictions that the p.Val43Leu change is unlikely to have a functional consequence, *ALS2CR12* seems a weak candidate for a BCC susceptibility gene.

Many variants are correlated with rs13014235 and they occur in a region of low recombination rate encompassing several genes (Fig. 1b). Two attractive candidate genes within the linkage disequilibrium block are *CASP10* and *CASP8*. These genes encode initiator caspases of the extrinsic pathways of apoptosis, controlled by death receptors from the tumour necrosis factor receptor superfamily^23^. To highlight this, we designated the locus 2q33 *CASP8-ALS2CR12*.

We observed a cluster of signals at 8q21 from variant alleles with protective effects and frequencies around 6.2%, typified by rs28727938 [G] (OR=0.70, *P*=2.1 × 10^−9^, Table 1, Fig. 1c). The signal peak is about 100 kb proximal to the zinc finger homeobox 4 (*ZFHX4*) gene and close to a related antisense non-coding RNA *ZFHX4-AS1*. Genotyping of non-Icelandic samples showed significant replication (OR=0.69, *P*=4.0 × 10^−4^) and a combined Icelandic and non-Icelandic result of OR=0.70, *P*=3.5 × 10^−12^ (Table 1).

In a peak at 10p14, the strongest signal originated from rs73635312 with the minor [A] allele showing a protective effect (OR=0.73, *P*=2.3 × 10^−13^) and a population frequency of 12.6% (Fig. 1d, Table 1). The peak occurs in the region of the lincRNAs *RP11-428L9.1* and *RP11-428L9.2*. The closest RefSeq gene to the peak is *GATA3*, although it is over 800 kb distal (Fig. 1d). The association was confirmed outside Iceland (OR=0.76, *P*=2.1 × 10^−4^). Combining the Icelandic and non-Icelandic data provided strong evidence of an association (OR=0.74, *P*=2.4 × 10^−16^, Table 1).

To determine whether any independent signals exist at the loci, we carried out an association analysis of all variants in each region ±500 kb surrounding each signal peak, conditioned on the effect of the top SNP. At 10p14 *GATA3*, we identified rs71483706 (Fig. 1d), whose minor allele conferred increased risk of BCC and had a frequency of about 32% (OR=1.17, *P*=2.8 × 10^−8^). In Iceland, rs71483706 retained a significant signal after adjustment for rs73635312 (*P*_adj_=4.2 × 10^−6^, OR_adj_=1.14). However, the association could not be replicated in the non-Icelandic population samples (*P*=0.31, OR=1.06). There was no evidence of secondary signals at any of the other three loci.

### Age at diagnosis

Sporadic BCC is predominantly a cancer of old age, with the majority of cases occurring in patients over the age of 60. However, in Gorlin syndrome, BCCs typically begin to appear in the early teens^24^. Accordingly, we investigated whether the variants we detected here and in earlier genome-wide association studies were associated with an earlier age at diagnosis of BCC (Supplementary Table 1). None of the four new variants reported here showed a significant association with age at diagnosis. The BCC variants reported previously had modest or no significant effects on age at diagnosis, though for the majority of variants the *β* estimates were negative. Therefore, it appears that, unlike the high-penetrance variants associated with Gorlin syndrome, common BCC predisposing variants have little impact on age at diagnosis.

### Fine mapping of variants to potentially functional sites

Because whole-genome sequencing was used for the detection and association testing of variants, we have a reasonably complete picture of the SNP and small indel variants present in Iceland down to a frequency of about 0.1%. At each locus, we evaluated every variant's candidature for pathogenic effect using two main criteria: first, we searched for variants that are correlated with the index SNP and whose association with BCC was statistically indistinguishable from the index SNP. Second, we looked for co-localization of these variants with biologically relevant landmarks (see Methods). The results are presented in Supplementary Data 1.

For the 2p24 *MYCN* locus, one variant besides the top SNP was highlighted by this process. This SNP, rs73217623, is highly correlated with the top SNP rs57244888 (*r*^2^=1.0, *P*_adj_=0.82). It is located in a DNaseI hypersensitivity site found in 54 cell types including normal human keratinocytes and in binding sites for seven transcription factors including CTCF in keratinocytes (Supplementary Data 1).

For the 2q33 *CASP8-ALS2CR12* locus, 195 variants were indistinguishable from the index SNP rs13014235 with respect to BCC risk, of which 32 had biologically relevant annotations. Of note, rs2349075 is highly correlated with rs13014235 (*r*^2^=0.99, *P*_adj_=0.75) and is located near an enhancer, which is active in keratinocytes, amongst other cell types. Keratinocytes and 75 other cell types show evidence of DNaseI hypersensitivity at this location and DNaseI hypersensitivity is correlated with expression of *CASP8*. Ten transcription factors are known to bind at the site including members of the jun/fos family. The rs2349075 variant is predicted to alter an AP-1-binding site (Supplementary Data 1).

At the 8q21 *ZFHX4* locus, two alleles of a multi-allelic indel that could not be distinguished from the top SNP rs28727938 had regulatory region annotations. However, neither they nor rs28727938 itself provided compelling evidence of a relevant function in keratinocytes (Supplementary Data 1).

For the 10p14 *GATA3* locus, we noticed that two correlated variants, SNP rs17413266 and indel rs144203968 (both having *r*^2^=0.90 versus rs73635312), occur in phylogenetically conserved regions which bind the GATA3 transcription factor. The SNP rs17413266 also has a Combined Annotation-Dependent Depletion Scaled C-score of 20.3, which is in the 98.8th percentile for an intergenic variant^25^ (Supplementary Data 1).

### Association with expression of CASP8 isoforms

**Figure 2:**
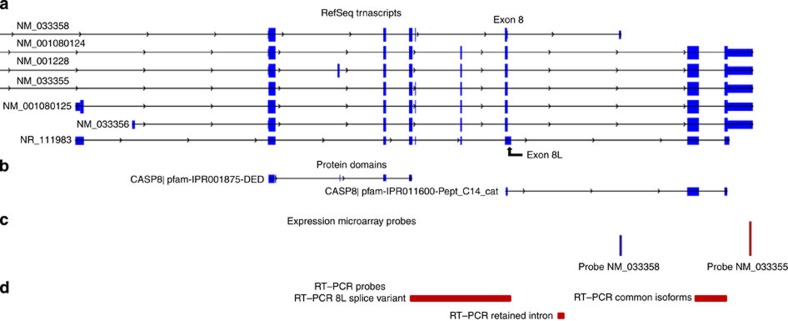
*CASP8*-coding exons, protein domains and locations of probes. (**a**) Coding exons of the major transcript variants. The positions of exon 8 and exon 8L are indicated. The transcript variant containing exon 8L is designated ‘NR_' because the exon 8L sequence contains a stop codon that may render this RNA species susceptible to nonsense-mediated decay. (**b**) Procaspase-8 protein domains corresponding to exons, from pfam: IPR001875-DED are the DED; IPR011600-Pept_C14_cat corresponds to the peptidase catalytic domains. (**c**) Location of the probe sequences used on the expression microarrays. The height of the bars corresponds to the relative levels of expression in blood. (**d**) Location of primer-amplicons used in RT–PCR.

We determined whether any of the BCC risk variants were associated with local differences in gene expression (*cis-*expression quantitative trait loci (eQTL)) by cross-referencing genotypes to RNA expression microarray data that we had derived previously from 1,001 blood and 673 adipose tissue samples^26^. No significant associations were seen for 2p24 *MYCN*, 8q21 *ZFHX4* or 10p14 *GATA3*. At 2q33, *CASP8* generates numerous splice variants (Fig. 2a). One probe on the microarrays, designated NM_033355, is in the 3′ UTR and captures all major *CASP8* isoform transcripts (Fig. 2c). A second probe, designated NM_033358, is unique to a small exon located between exons 8 and 9 of the major transcripts (exon numbering is based on NM_001228). The NM_033358 transcript encodes caspase-8 isoform E, which contains the death effector domains (DED) but lacks the catalytic domains of caspase-8 (Fig. 2b). A transcript with an extended exon 8, called exon 8L (NR_111983) has the capacity to encode a similar DED-only isoform because the 8L extension contains an in-frame stop codon. However, splicing of exon 8L to downstream exons may target the transcript for nonsense-mediated decay^27^,^28^,^29^,^30^. Evidence for the existence in *vivo* of DED-only caspase-8 isoforms and their potential inhibitory effects on apoptosis has been controversial^28^,^29^,^31^,^32^,^33^,^34^.

**Figure 3:**
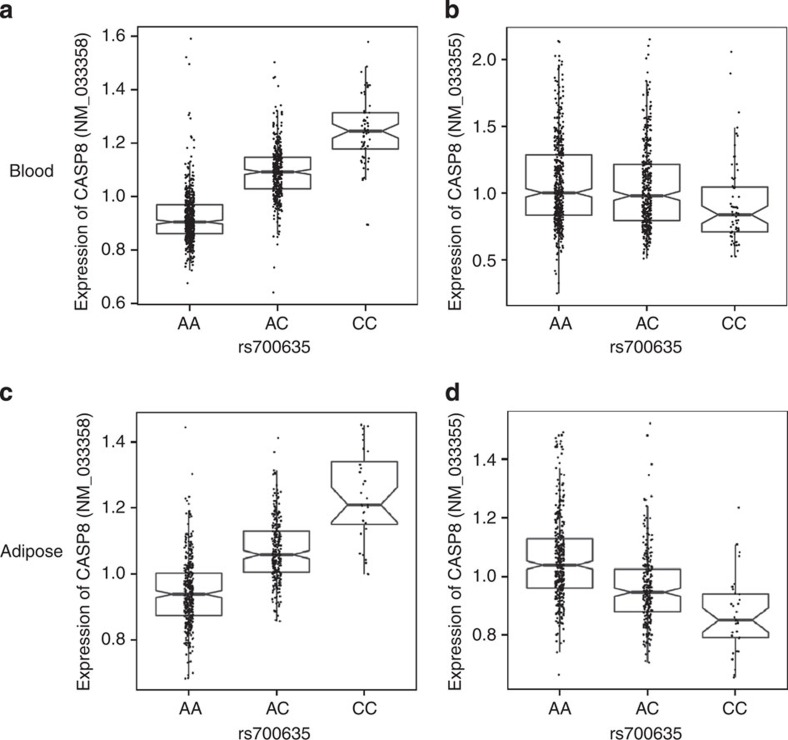
RNA expression microarray data for *CASP8* transcript variants, stratified by genotype of BCC risk variant rs700635. RNA from blood and adipose tissue was hybridized to Agilent expression microarrays containing probes for the major *CASP8* transcript variants (NM_033355) and the variant encoding isoform E (NM_033358). Normalized expression levels were stratified by genotype of variant rs700635, allele [C] being the risk allele for BCC. Effects and *P* values were determined using multivariable linear regression. The box plots show median, 25th and 75th percentiles (boxes) and 5th and 95th percentiles (vertical lines). Individual points for each sample are also plotted. (**a**) Expression of the isoform E transcript variant NM_033358 in blood. eQTL result coded to rs700635[C] is β=0.0698, *P*=1.2 × 10^−119^. (**b**) Expression of the major transcript variants NM_033355 in blood. *β*=−0.0195, *P*=0.0076. (**c**) Expression of the isoform E transcript variant NM_033358 in adipose tissue. *β*=0.0571 *P*=3.7 × 10^−63^. (**d**) Expression of the major transcript variants NM_033355 in adipose tissue. *β*=−0.0436, *P*=7.9 × 10^−22^.

We found that the BCC risk allele rs13014235[C] is associated with a significant increase in abundance of transcripts containing the NM_033358 probe sequence in both blood (*P*=1.9 × 10^−39^) and adipose tissue (*P*=3.1 × 10^−19^). This is accompanied in adipose by a modest reduction in abundance of the major *CASP8* transcripts as detected by probe NM_033355 (Supplementary Fig. 3). In fact, the expression of the isoform E transcript variant NM_033358 correlated negatively with expression of the major *CASP8* transcript variants regardless of genotype (*P*=2.1 × 10^−18^, Supplementary Fig. 4). A search of the surrounding genomic region identified rs700635[C], which is a member of the class of variants that could not be distinguished from rs13014235 with respect to BCC risk and which exhibited the strongest association with expression of the isoform E variant NM_033358 (*β*=0.0698, *P*=1.2 × 10^−119^ in blood RNA, Fig. 3). It also associated with a modest decrease in expression of the major *CASP8* transcripts and had a similar pattern in adipose tissue.

**Figure 4:**
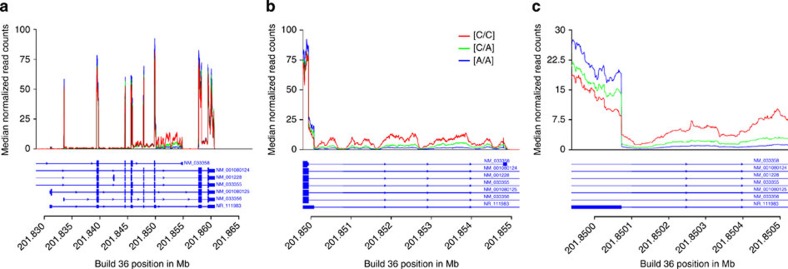
Carriers of the rs700635[C] BCC risk allele show preferential retention of *CASP8* intron 8. RNA-seq data was obtained from blood from genotyped donors. These were *n*=24 rs700635[C/C] homozygotes (shown in red), *n*=117 rs700635[C/A] heterozygotes (shown in green) and *n*=114 rs700635[A/A] homozygotes (shown in blue). The *x* axis is the genomic position in Mb (hg18/Build36). The *y* axis is, for each genotypic group, the median count of normalized reads (normalized for each individual to the total number of aligned reads). The structure of the RefSeq transcript variants is shown beneath the graphs. (**a**) The genomic region covering the coding exons of *CASP8*. (**b**) Zoom showing the exon 8, exon 8L region extending through intron 8 to the minor exon targeted by the NM_033358 probe. (**c**) Zoom showing the splice junction between exon 8L and intron 8.

Given the biological importance of *CASP8*, we initiated a more detailed investigation of its expression. We generated blood RNA-seq data from an independent set of 261 genotyped individuals. For rs700635[C], we confirmed that RNA abundance in the NM_033358 probe region is increased in risk allele carriers. However, RNA-seq revealed that the entire intron 8 sequence between exon 8L and the probe region is preferentially retained in carriers (Fig. 4a,b). This coincides with the reduced expression of the major exons of the *CASP8* gene in association with rs700635[C] (*β*=−0.65 *P*=1.7 × 10^−12^, Supplementary Table 2). Reverse transcription–PCR (RT–PCR) of 76 blood RNA samples (using probes as shown in Fig. 2d) reconfirmed these findings (Supplementary Fig. 5).

Retention of intron 8 appears to result from failures to recognize splice donors at the 3′ ends of exon 8 and exon 8L, with the exon 8L donor being ignored preferentially in rs700635[C] carriers (*P*=6.2 × 10^−22^, Supplementary Fig. 6). Some intron 8 reads had paired ends spliced from upstream exons, which helped to establish the sense strand polarity of the retained intron transcripts. We also saw reads indicating that exon 8 (but not exon 8L) can splice to the NM_033358 probe region. However, the efficiency of this splice did not vary by genotype (*P*=0.89, Supplementary Fig. 6). Of the 195 variants that were indistinguishable from the index SNP with respect to BCC risk, none occurred at the exon 8 or 8L splice donor sites or the exon 9 splice acceptor. Limited data are available from intron 8 beyond the probe region, as there is very restricted read coverage 3′ to the NM_033358 probe in both RNA and DNA sequences. Nevertheless, we did detect RNA-seq read pairs crossing the intron 8–exon 9 boundary, suggesting that the entirety of intron 8 fails to splice out in association with rs700635[C].

To investigate whether similar phenomena occur at *CASP8* in epidermis, we obtained RNA-seq and genotype data from the GTEx project^35^ and analysed them using the same methods as described above (Supplementary Fig. 7). In sun-exposed skin, we again found that intron 8 including the NM_033358 probe sequence is expressed preferentially in association with the rs700635[C] BCC risk allele (*β*=1.17, *P*=1.8 × 10^−24^ for intron 8, Supplementary Table 2). The major *CASP8* isoform variants were also expressed at reduced levels in BCC risk allele carriers in sun-exposed skin (*β*=−0.64, *P*=7.2 × 10^−9^), in agreement with our findings in blood and adipose tissue. We did notice that preferential expression from rs700635[C] extended to sequences in exon 8 and intron 7, which was not seen by us in blood and remains unexplained (Supplementary Fig. 7). A search of the GTEx sun-exposed skin RNA-seq data for eQTLs related to the lead SNPs at 2q24 *MYCN*, 8q21 *ZFHX4* and 10p14 *GATA3* yielded no significant associations.

### Risk variants for other cancers at *CASP8*

*CASP8* has been implicated in several cancer types both through germline variants and somatic mutations. The p.Asp302His germline variant rs1045485[C] has been associated with protection from breast cancer^36^,^37^. We confirmed this association to nominal significance in the Icelandic population (OR=0.93, *P*=0.031). However, we saw no effect of the allele on BCC risk (OR=1.04, *P*=0.31, 95% confidence interval (CI)=0.97–1.11). *CASP8* was also suggested as the likely candidate gene underlying the effect of a melanoma susceptibility variant in *ALS2CR12* (rs13016963) (ref. 38). In our data, we could not see evidence of this melanoma association (OR=1.04, *P*=0.40, 95% CI=0.96–1.12). There was a nominal association of rs13016963 with BCC (OR=1.08, *P*=0.0036) but it was fully accounted for by rs13014235, the index BCC SNP at the locus (*P*_adj_=0.79). A common chronic lymphocytic leukaemia risk variant^39^ did not show a BCC association (rs3769825, OR=1.03, *P*=0.35, 95% CI=0.97–1.08 ), nor did the so-called −652 6N del *CASP8* promoter deletion (rs3834129) that has been controversially implicated in susceptibility to a variety of cancers^40^,^41^ (OR=0.95, *P*=0.069, 95% CI=0.91–1.00). Moreover, we have not as yet detected a significant association between the BCC index SNP rs13014235 and any other type of cancer documented by the Icelandic Cancer Registry. Recently, Lin *et al.*^42^ reported an association between rs1830298 in *ALS2CR12* and breast cancer. That variant is highly correlated with the strongest *CASP8* eQTL variant we report (rs700635, *r*^2^=1.0). We confirmed the association with breast cancer for rs700635 (OR=1.08, *P*=0.0053), but did not see a significant association with rs13014235 (OR=1.03, *P*=0.27). This suggests a complex pattern of cancer risk associations at the locus, reminiscent of the *TERT-CPTLM1L* locus previously identified in our BCC genome-wide association study^17^.

## Discussion

We report here on the discovery of four new susceptibility loci for BCC. At the 2q24 locus, *MYCN* is a known oncogene with involvement in neuroblastoma and medulloblastoma^43^,^44^; however, it has not been implicated previously in BCC. Similarly, there are no reports of an involvement of *ZFHX4* in cancer predisposition. *CASP8* and *GATA3*, on the other hand, do have known associations with both oncogenesis and epidermal development.

Following death receptor ligation, procaspase-8 is recruited to the cytoplasmic death-inducing signalling complex (DISC) through interactions involving its DED. Recruitment of procaspase-8 to the DISC leads to its dimerization and activation through induced proximity. Full activation of caspase-8 results from cleavage of the catalytic domains from the DED and release of the dimerized catalytic domains from the DISC^23^. Our findings indicate that in BCC risk allele carriers there is a preferential retention of the intron following exons 8 and 8L. RNA species with this structure have the capacity to encode caspase-8 isoforms, which contain the DED but lack the catalytic domains. Conceptually, an isoform with this structure has the potential to inhibit caspase-8 signalling by interfering with the binding of full-length procaspase-8 to the DISC. Indeed some (but not all) investigators have observed anti-apoptotic effects resulting from the expression of DED-only caspase-8 isoforms^28^,^33^,^34^. If the RNA species run through to exon 9, splicing of exon 9 to exon 10 could, however, render the transcripts susceptible to nonsense-mediated decay. Coincident with the intron retention is a reduction in overall expression of the major transcript variants of *CASP8*. Combined, these factors could suppress caspase-8 signalling in risk allele carriers.

One predictable outcome of reduced caspase-8 signalling might be a failure of incipient tumour cells to undergo apoptosis. During normal epidermal development there does not, however, seem to be an involvement of activated pro-apoptotic caspases^45^. Conditional knockout mice where *CASP8* is specifically disabled in epidermal keratinocytes develop a lethal neonatal skin inflammation and hyperplasia^46^,^47^. Double knockout of *CASP8* and the necroptosis-associated kinase *RIPK3* rescues mice from neonatal lethality, skin inflammation and hyperplasia^23^,^48^. This suggests that *CASP8* normally functions to suppress necroptosis and consequent inflammation in epidermis. It is therefore possible that an inhibition of caspase-8 signalling in BCC risk allele carriers could act to promote necrosis and inflammation, thereby generating a tumour-promoting environment within the epidermis.

We caution that while the effect of rs700635 on the expression of *CASP8* is robustly proven and the SNP is associated with risk of BCC (OR=1.15, *P*=1.0 × 10^−6^ in Iceland), rs700635 is not the most strongly associated BCC risk variant at the locus. Although rs700635 is included on the list of potentially causative variants, it is not extremely well correlated with the index SNP rs13014235 (*r*^2^=0.48) and rs13014235 retains a nominally significant BCC association signal when adjusted for the effect of rs700635 (*P*_adj_=0.022, Supplementary Data 1). The relationship between BCC risk and *CASP8* transcript variant expression therefore remains tentative and merits further study. There are genes besides *CASP8* at the 2q33 locus that should be considered. Like *CASP8*, *CASP10* encodes an extrinsic apoptosis initiator caspase. It is less well characterized, in part, because it has no counterpart in mice. Somatic mutations in *CASP10* have been found in gastric cancer and non-Hodgkin's lymphoma^49^,^50^. Also, in the region is *STRADB*, encoding a pseudokinase that complexes with STK11 and may have anti-apoptotic functions^51^.

The closest gene to the 10p14 signal is *GATA3*, encoding a C2H2 zinc finger type transcription factor that binds to sequences resembling ^A^/_T_GATA^A^/_G_ in the proximity of target genes, where it can activate or repress transcription. *GATA3* is involved in lineage specification and differentiation in a variety of settings, including T cells, mammary gland epithelia and skin^52^. In skin, *GATA3* is involved in lineage determination of the inner root sheath of the hair follicle. *GATA3*-knockout mice and conditional knockouts, where *GATA3* is specifically disrupted in the skin, show a failure of correct specification of inner root sheath cells and consequent disruption of hair follicle development^53^,^54^. In normal interfollicular skin, *GATA3* is expressed in the suprabasal layer and persists throughout the differentiating layers^54^,^55^. *GATA3* knockouts show a hyperplasia of the basal layer, disruption of the stratified epithelial differentiation programme and skin barrier defects^53^,^54^,^56^. Taken together with our association data these reports suggest that, in humans, variants at the *GATA3* locus may affect the specification and differentiation programme of the epidermis.

Although *GATA3* is the nearest gene to the variants we describe at 10p14, the signal peak is nevertheless a substantial distance (>800 kb) downstream of the gene. Analysis of ENCODE data revealed two highly correlated variants (rs17413266 and rs144203968) that occur in ChIP-Seq verified GATA3 binding sites. Ouyang *et al.*^57^ have shown that in CD4^+^ T cells, GATA3 initiates an auto-activation feedback loop that promotes ongoing *GATA3* expression and stabilizes Th2 lineage commitment. The molecular details of this auto-activation have never been worked out and it is not known if a similar feedback loop occurs in differentiating epidermis.

In summary, we have found four new BCC susceptibility loci. While the mechanisms at two of the loci remain cryptic, we provide testable hypotheses for roles of *CASP8* and *GATA3* variants in epidermal carcinogenesis.

## Methods

### Subjects

Approval for the Icelandic study was granted by the Icelandic National Bioethics Committee and the Icelandic Data Protection Authority. All participants provided informed consent. Affected individuals were identified through the Icelandic Cancer Registry (ICR), which has maintained records of BCC diagnoses since 1981. The records contained only cases of histologically verified BCC, sourced from all the pathology laboratories in the country that deal with these lesions. Icelandic controls consisted of individuals selected from other ongoing association studies at deCODE and who did not have a diagnosis of BCC recorded in the ICR. Median age at diagnosis for ICR notified cases was 68 years (range 10–104). All subjects were of European ancestry. Compared with our most recent prior publication on BCC^16^, the present study adds 364 new BCC cases and 156,950 new controls. Of these, 254 cases and 16,944 controls were typed on Illumina chips resulting in a total of 17,198 new chip genotypes being available to the study. There was also an increase in the numbers of close relatives for whom genotype probabilities could be used: 110 additional BCC cases and 140,006 controls.

Eastern European BCC cases were recruited from all general hospitals in three study areas in Hungary, two in Romania and one in Slovakia. Cases were identified based on histopathological examinations by pathologists. The median age at diagnosis was 67 years (range 30–85). Controls were recruited from the same hospitals from surgical, orthopaedic and trauma patients. Controls were thoroughly screened and individuals with past or current BCC, malignant disease, cardiovascular disease or diabetes were excluded. All subjects were of self-reported European ancestry. The study was approved by the Ethical Committee of the National Health Research Council, Hungary. In Romania, ethical aspects of the study were approved by Environmental Health Centre and Public Health Department. In Slovakia, ethical aspects of the study were approved by the Ethical Committee of State Health Institutes.

*Spain Valencia*. Participants were recruited from the outpatient dermatology clinics of the Instituto Valenciano de Oncología in Valencia, Spain, starting from May 2003. Cases were patients with histologically proven BCC presenting with superficial or nodular lesions of <1 cm in diameter. Clinical and pathological data from these patients are prospectively collected by medical history review, personal interview and clinical examination by an expert dermatologist. Immunocompromised patients were excluded as were those with any autoimmune disease, hereditary disorders that include the presence of BCC (Gorlin syndrome, xeroderma pigmentosum) and epidermodysplasia verruciformis. Controls were disease free and ethnically matched healthy subjects recruited at the Transfusion Centre of Valencia. Phenotypic characteristics of controls were obtained by a self-administered structured questionnaire. Prospective controls reporting past or present BCC were excluded. All subjects were of self-reported European ancestry. All patients in the study had signed an informed consent and the study protocol was approved by the ethics boards of the Instituto Valenciano de Oncología and the Transfusion Centre of Valencia.

*Spain Zaragoza*. BCC cases were recruited from the Oncology Department of Zaragoza University Hospital starting from September 2007. Individuals with histologically proven invasive BCC were eligible to participate in the study. The median time interval from BCC diagnosis to collection of blood samples was 14 months (range 1–53 months). Median age at diagnosis was 69 years (range 21–91). Controls were recruited from individuals who attended the Zaragoza University Hospital for diseases other than cancer. Prospective control subjects were questioned by the recruiter about past or present malignant disease, including BCC, and those with a positive history were excluded. All subjects were of self-reported European ancestry. All subjects in the study had signed an informed consent and the study protocol was approved by the Zaragoza University Hospital ethics board.

*Denmark*. Subjects were participating in the ‘Diet, Cancer and Health' study, which is a prospective study of 57,053 individuals recruited at two centres in Denmark between December 1993 and May 1997 (ref. 58). Subjects were monitored during the follow-up period and BCC cases were identified through linkage to entries in the Danish Cancer Registry. Each case identified was matched to one control from the Diet, Cancer and Health cohort based on gender, age on study entry and age at diagnosis of the case. Median age at diagnosis was 59 (range 50–68). In addition to extensive diet and lifestyle questions, subjects answered questions regarding their skin sensitivity to sun, tanning during summer, presence of nevi and presence of freckles. All subjects were of self-reported European ancestry. The study ‘Diet, Cancer and Health' has been approved by the regional Ethical Committees on Human Studies in Copenhagen and Aarhus, and by the Danish Data Protection Agency. Informed consent was obtained from all participants to search information from medical registers including the Danish Cancer Register.

### Illumina SNP chip genotyping

The Icelandic chip-typed samples were assayed with the Illumina HumanHap300, HumanHapCNV370, HumanHap610, 1M, or Omni-Quad bead chips at the deCODE Genetics facility. All samples with a call rate of <97% were removed from the analysis. SNPs were excluded if they had (i) a yield lower than 95%, (ii) a minor allele frequency of <0.01 in the population, (iii) an excessive deviation from the Hardy–Weinberg equilibrium (*P<*10^−6^), (iv) an excessive inheritance error rate (>0.001) or (v) if there was a substantial difference in allele frequency between chip types (in which case the SNP was removed from a single chip type if that resolved the difference, but if it did not then the SNP was removed from all chip types). None of the SNPs listed in Table 1 showed a significant association with chip type (*P* values were between 0.11 and 0.77, tested using logistic regression).

### Whole-genome sequencing imputation and association testing

In this study, we sequenced 2,636 Icelanders to a median depth of 20 × . Long-range haplotype phasing and genealogy-based calculation of genotype probabilities^15^,^19^,^20^,^21^ of the variants identified by sequencing allowed us to perform 24,988,228 tests of association; 19,543,184 tests for SNPs and 5,445,044 tests for small indels (≤60 bp). We tested only alleles that could be imputed with an information value ≥0.8, where the informativeness of genotype imputation was estimated by the ratio of the variance of imputed expected allele counts to the variance of the actual allele counts, as described previously^15^. This permitted us to test variants down to a minor allele frequency of ∼0.1%. In association testing, we tested each allele against all other alleles at that position combined. Thus, for biallelic variants, this corresponds to one test, whereas for multi-allelic variants this corresponds to *n* tests, where *n* is the number of qualifying alleles. All SNP and indel locations are given in NCBI hg18/Build 36 coordinates unless otherwise specified. Indels are designated as chr:position:I. Multi-allelic variants are designated as chr:position:0:allele, for example, ‘chr8:77605042:0:GAA'.

We imputed the genotypes of 4,572 Icelanders with BCC (cases) and 266,358 controls. The BCC cases were comprised of 2,980 individuals who had been directly typed on Illumina SNP genotyping microarray chips and 1,592 close relatives of chip-typed individuals. The controls were comprised of 87,820 chip-genotyped individuals and 178,538 close relatives of chip-typed individuals. For the HumanHap series of chips, 304,937 SNPs were used for long-range phasing, whereas for the Omni series of chips 564,196 SNPs were used. An initial imputation step was carried out on each chip series separately to create a single harmonized, long-range phased genotype data set consisting of 707,525 SNPs. In this first step, sharing between individuals with the same long (>10 cM) haplotypes was used to fill in genotypes of SNPs not present on both chip platforms. So, for example, if a parent was genotyped with a HumanHap series chip and an offspring with an Omni chip, then the alleles of the transmitted chromosome can be inferred for both individuals. Since chip genotypes are available for a large fraction of the Icelandic population (which currently numbers 320,000), this process is quite complete with only 1% of alleles remaining unknown. Subsequently, this genotype data set was used in the second step of imputing the full set of variants which was used in the 24,988,228 tests of association. Association testing was performed using logistic regression, treating disease status as the response and genotype counts as covariates. Other available individual characteristics that correlate with disease status were also included in the model as nuisance variables. These characteristics were gender, county of birth, current age or age at death (first- and second-order terms included), blood sample availability for the individual and an indicator function for the overlap of the lifetime of the individual with the timespan of phenotype collection^21^. Correction for familial relatedness was carried out using genomic control and *P* values were adjusted accordingly (*λ*=1.2374). Tests for heterogeneity were performed by comparing the null hypothesis of the effect being the same in all populations to the alternative hypothesis of each population having a different effect, using a likelihood ratio test. For conditional analyses, the allele count of each individual was given as a covariate in the logistic regression. All *r*^2^ values quoted refer to the Icelandic population and were determined using long-range haplotype phasing and imputation.

### Assessment for overlap with regulatory regions

To identify a set of BCC-associated variants that might have regulatory effects, we took the index variant at each locus and adjusted its association signal in turn for the effect of each other variant within ±500 kb. If the resulting *P*_adj_ was >0.001, we considered that the tested variant could not safely be distinguished from the index variant and should therefore be evaluated for potential regulatory and pathogenic effect (irrespective of its *r*^2^ value relative to the index variant). This resulted in sets containing 45 variants for 2p24 *MYCN*, 41 variants for 8q21 *ZFHX4*, 52 variants for 10p14 *GATA3* and 195 variants for the 2q33 *CASP8-ALS2CR12* locus. Note that for this analysis, we used only variants that were detected by the whole-genome sequencing and which could be imputed with stringent information values ≥0.9 (ref. 15).

For each variant in the sets thus identified, we searched for overlaps with known regulatory regions as follows: first, we used ENSEMBL to determine whether the variant had been assigned a regulatory region ENSR number. Then we examined the ENCODE data and recorded any evidence of ChIP-Seq transcription factor binding, DNaseI hypersensitivity sites, enhancer and promoter chromatin segmentation states^59^,^60^,^61^. We also recorded enhancer and promoter chromatin segmentation states using the 25 state HMM from the Roadmap consortium^62^. Then we looked for correlations between DNaseI hypersensitive sites and local gene expression using results described by Sheffield *et al.*^63^ We examined SiPhy and GERP conservation scores and the Combined Annotation-Dependent Depletion Scaled C-score^25^,^64^,^65^. We also viewed the Factorbook and HaploReg_v2 data^66^,^67^ ( http://www.broadinstitute.org/mammals/haploreg/haploreg.php) to search for potential changes in transcription factor-binding motifs but we did not annotate the variants with these data unless there was ChIP-Seq evidence of the cognate transcription factor binding at that location. To narrow down the variants for scrutiny at each locus, we pass-filtered the variants on (a) being the index BCC-associated marker at the locus or (b) presence of an ENSR number or (c) a reference to normal human keratinocytes (NHEK) or primary foreskin keratinocytes (PFK) in the annotations derived above. After filtration, there remained 2 variants at *MYCN*, 3 at *ZFHX4*, 2 at *GATA3* and 32 at *CASP8-ALS2CR12*. We manually checked the data for each of these variants in the UCSC GRCh37/hg19 Test Genome Browser and by using HaploReg_v2. The results were tabulated using a style based on the HaploReg_v2 presentation (Supplementary Data 1). We also manually checked the annotations for the unfiltered variants with *P*_adj_>0.001 from each locus. This resulted in the identification of two additional variants at the *GATA3* locus that are located in GATA3 transcription factor-binding sites but did not meet the pass-filter qualifications (see main text and Supplementary Data 1).

### Gene expression microarrays

Samples of RNA from human peripheral blood (*N*=1001) and adipose tissue (*N*=673) were hybridized to Agilent Technologies Human 25K microarrays^26^. We quantified the expression changes between two samples as the mean logarithm (log_10_) expression ratio (MLR) compared with a reference pool RNA sample. In comparing the expression levels between groups of individuals with different genotypes, we denoted the expression level for each genotype as 10^(average MLR)^, where the MLR is averaged over individuals with the particular genotype. We determined the s.e. and significance by regressing the MLR values against the number of risk alleles carried. We took into account the effects of age, gender and differential cell type count in blood as explanatory variables in the regression. *P* values were adjusted for familial relatedness of the individuals by simulation. No significant eQTLs were associated with the risk alleles at the 2p24 *MYCN*, 8q21 *ZFHX4* or 10p14 *GATA3* loci.

### RNA-seq

For preparation of poly-A complementary DNA (cDNA) sequencing libraries, the quality and quantity of isolated total RNA from 261 peripheral blood samples was assessed using the Total RNA 6000 Nano chip for the Agilent 2100 Bioanalyzer. Samples were from different individuals than those who had been sampled for the gene expression microarray studies described above. cDNA libraries derived from Poly-A mRNA were generated using Illumina's TruSeq RNA Sample Prep Kit. In brief, Poly-A mRNA was isolated from total RNA samples (1–4 μg input) using hybridization to Poly-T beads. The Poly-A mRNA was fragmented at 94 °C, and first-strand cDNA was prepared using random hexamers and the SuperScript II reverse transcriptase (Invitrogen). Following second-strand cDNA synthesis, end repair, addition of a single A base, adaptor ligation, AMPure bead purification and PCR amplification, the resulting cDNA was measured on a Bioanalyzer using the DNA 1000 Lab Chip.

Samples for sequencing were clustered on to flowcells using Illumina's cBot and the TruSeq PE cluster kits v2, respectively. Paired-end sequencing (2 × 76 cycles) was performed with either GAIIx instruments using the TruSeq SBS kits v5 from Illumina or HiSeq 2000 instruments using TruSeq v3 flowcells/SBS kits. In some instances, the read length was 2 × 50 or 2 × 101 cycles. Approximately, 125–175 million forward reads (250–350M total reads) were sequenced per sample.

RNA sequencing reads were aligned to Homo sapiens Build 36 with TopHat^68^ version 1.4.1 with a supplied set of known transcripts in GTF format (RefSeq hg18; Homo sapiens, NCBI, build36.3). TopHat was configured such that it attempts first to align reads to the provided transcriptome then, for reads that do not map fully to the transcriptome, it attempts to map them onto the genome. Read mapping statistics used for read count normalization were calculated using the CollectRnaSeqMetrics tool in Picard version 1.79 ( http://picard.sourceforge.net/command-line-overview.shtml#CollectRnaSeqMetrics). Once read alignment by TopHat was finished, gene expression estimation was done using Cufflinks^68^ version 1.3.0 for the same set of transcripts used in the read alignment by TopHat.

For analysis of RNA-seq samples, the normalized read count was determined for each bp location as the number of reads covering the base/the total number of aligned reads in the individual in billions. Then the median normalized read count for each genotype group was determined for each bp location and plotted graphically. To quantify the levels of expression in different regions, the median of normalized read counts over the genomic segment in question was determined for each individual. We then did a logarithmic transformation of the normalized read counts and standardized them so that the response variable had a mean 0 and s.d. of 1. We determined the effect and significance by regressing the transformed read counts against the number of risk alleles carried. Effect sizes (*β*) are given in s.d. units.

Data from the GTEx Release phs000424.v4.p1 from 17.01.2014 were downloaded from the GTEx consortium^35^ ( http://www.gtexportal.org/home/) *via* dbGAP. Analysis was conducted using the provided aligned read and genotype data as described above for deCODE data. One exception was the integrated CASP8 whole-gene read count by genotype calculation for skin (Supplementary Table 2) which was taken directly from the GTEx portal and employed their methods (see URLs).

### RT–PCR

We converted total RNA (from a subset of 76 of the blood RNA samples that were used for the expression microarrays) to cDNA using the High Capacity cDNA Archive Kit (Applied Biosystems), primed with random hexamers. We designed assays spanning the exon–intron junction between exons 8 and 9 (RT–PCR common isoforms, Fig. 2, F-primer: 5′-tatatcccggatgaggctga-3′, R-primer: 5′-tcaatcagaagggaagacaagtt-3′), spanning from exon 6 to a sequence unique to exon 8L (RT–PCR 8L splice variant, Fig. 2, F-primer: 5′-gcctgctgaagataatcaacg-3′, R-primer: 5′-gaaacatggcccttttggta-3′) and contained within intron 8, 5′ to the NM_033358 probe sequence (RT–PCR retained intron, Fig. 2, F-primer: 5′-tcaatttatgaatggacaaccaa-3′, R-primer: 5′-cctctctttcgtattagagcctttc-3′). Primer sequences are available on request. We carried out quantitative PCR according to the manufacturer's instructions on an ABI Prism 7900HT Sequence Detection System. We assessed differences in mean relative RNA abundance between genotypic groups by regression, as described above for expression microarrays.

## Author contributions

S.N.S., H.H., S.A.G., G.T., F.Z., P.S., A.K., D.F.G., U.T., J.H.O. and K.S. designed the study and interpreted the results. S.N.S., B.S., K.R.B., K.T., R.R., V.F., C.C., Y.G., M.G., D.P., O.S., P.R., E.G., K.K., B.A.N., A.T., K.O., J.G.J., L.T., T.R., U.V., R.K., E.N., J.I.M. and J.H.O carried out the subject ascertainment and recruitment. S.N.S., H.H., G.T., F.Z., A.S., H.J., A.M.K., H.S., G.M., O.T.M., T.R. and U.T. performed the sequencing, genotyping and expression analyses. S.N.S., H.H., S.A.G., G.T., F.Z., A.S., B.K., P.S., G.M., B.V.H., A.K. and D.F.G. performed the statistical and bioinformatics analyses. S.N.S., H.H., S.A.G., G.T., F.Z. and K.S. drafted the manuscript. All authors contributed to the final version of the paper. Principle collaborators for the case–control population samples were B.S. and J.H.O. (Iceland), U.V. (Denmark), R.K. (Eastern Europe), E.N. (Valencia, Spain) and J.I.M (Zaragoza, Spain).

## Additional information

**How to cite this article**: Stacey, S. N. *et al.* New basal cell carcinoma susceptibility loci. *Nat. Commun.* 6:6825 doi: 10.1038/ncomms7825 (2015).

## Supplementary Material

Supplementary Information Supplementary Figures 1-7 and Supplementary Tables 1-2

Supplementary Data 1 The co-localization to biologically relevant landmarks of variants whose associations are statistically indistinguishable from the index SNP at each locus

## Figures and Tables

**Table 1:** Association of SNPs at four loci with basal cell carcinoma.

**SNP**	**Allele**	**Chr**	Position*	**Locus**	**Sample set**	**Number of cases**†	**Number of controls**‡	**Frequency in controls**	**OR**	**95%CI**	***P*****value**§	***P***_**het**_ **value**||
rs57244888	C	2	16,325,626	2p24 MYCN	Iceland	4,572	266,358	0.102	0.77	(0.70, 0.84)	1.4 × 10^−8^	
					Spain Zaragoza	316	1,912	0.128	0.76	(0.58, 1.01)	0.059	
					Spain Valencia	334	1,989	0.112	0.73	(0.55, 0.97)	0.031	
					Eastern Europe	521	528	0.126	0.71	(0.54, 0.94)	0.017	
					Denmark	303	309	0.094	0.74	(0.49, 1.11)	0.15	
					**Combined Non-Icelandic**	**1,474**	**4,738**		**0.74**	(**0.63, 0.86)**	**6.9 × 10**^−**5**^	**0.99**
					**All Combined**	**6,046**	**271,096**		**0.76**	(**0.70, 0.82)**	**4.7 × 10**^−**12**^	**0.98**
rs13014235	C	2	201,923,737	2q33 CASP8-ALS2CR12	Iceland	4,572	266,358	0.456	1.15	(1.09, 1.21)	1.6 × 10^−7^	
					Spain Zaragoza	312	1,883	0.396	1.16	(0.97, 1.38)	0.094	
					Spain Valencia	334	1,945	0.399	1.14	(0.96, 1.35)	0.12	
					Eastern Europe	525	526	0.427	1.14	(0.95, 1.36)	0.15	
					Denmark	297	303	0.386	1.18	(0.93, 1.50)	0.18	
					**Combined Non-Icelandic**	**1,468**	**4,657**		**1.15**	(**1.05, 1.26)**	**0.0027**	**1.00**
					**All Combined**	**6,040**	**271,015**		**1.15**	(**1.10, 1.20)**	**1.5 × 10**^−**9**^	**1.00**
rs28727938	G	8	77,641,094	8q21 ZFHX4	Iceland	4,572	266,358	0.062	0.70	(0.62, 0.79)	2.1 × 10^−9^	
					Spain Zaragoza	313	1,910	0.059	0.66	(0.43, 1.00)	0.050	
					Spain Valencia	331	1,988	0.065	0.65	(0.44, 0.97)	0.037	
					Eastern Europe	525	526	0.061	0.72	(0.48, 1.09)	0.12	
					Denmark	302	309	0.081	0.72	(0.46, 1.12)	0.15	
					**Combined Non-Icelandic**	**1,471**	**4,733**		**0.69**	(**0.56, 0.85)**	**4.0 × 10**^−**4**^	**0.98**
					**All Combined**	**6,043**	**271,091**		**0.70**	(**0.63, 0.77)**	**3.5 × 10**^−**12**^	**0.99**
rs73635312	A	10	8,976,004	10p14 GATA3	Iceland	4,572	266,358	0.126	0.73	(0.68, 0.80)	2.3x10^−13^	
					Spain Zaragoza	313	1,904	0.120	0.81	(0.61, 1.07)	0.14	
					Spain Valencia	330	1,993	0.114	0.65	(0.48, 0.88)	0.0045	
					Eastern Europe	526	528	0.153	0.72	(0.56, 0.93)	0.013	
					Denmark	306	306	0.142	0.93	(0.67, 1.29)	0.68	
					**Combined Non-Icelandic**	**1,475**	**4,731**		**0.76**	(**0.66, 0.88)**	**2.1 × 10**^−**4**^	**0.41**
					**All Combined**	**6,047**	**271,089**		**0.74**	(**0.69, 0.80)**	**2.4 × 10**^−**16**^	**0.54**

Chr, chromosome; CI, confidence interval; OR, odds ratio; SNP, single-nucleotide polymorphism.

^*^NCBI hg18 Build 36.

^†^For Iceland, this is the total number of cases used for association testing, including 2,980 chip genotyped and 1,592 related individuals.

^‡^For Iceland, this is the total number of controls used, including 87,820 chip genotyped and 178,538 related individuals.

^§^Association testing was done using logistic regression.

^||^Likelihood ratio test for heterogeneity.
