# Supplementary material for: New basal cell carcinoma susceptibility loci
Source: Nat Commun. 2015 Apr 9;6:6825. doi: 10.1038/ncomms7825 (PMC4403348; doi:10.1038/ncomms7825)
Supplement: Supplementary Information — Supplementary Figures 1-7 and Supplementary Tables 1-2 [file ncomms7825-s1.pdf]

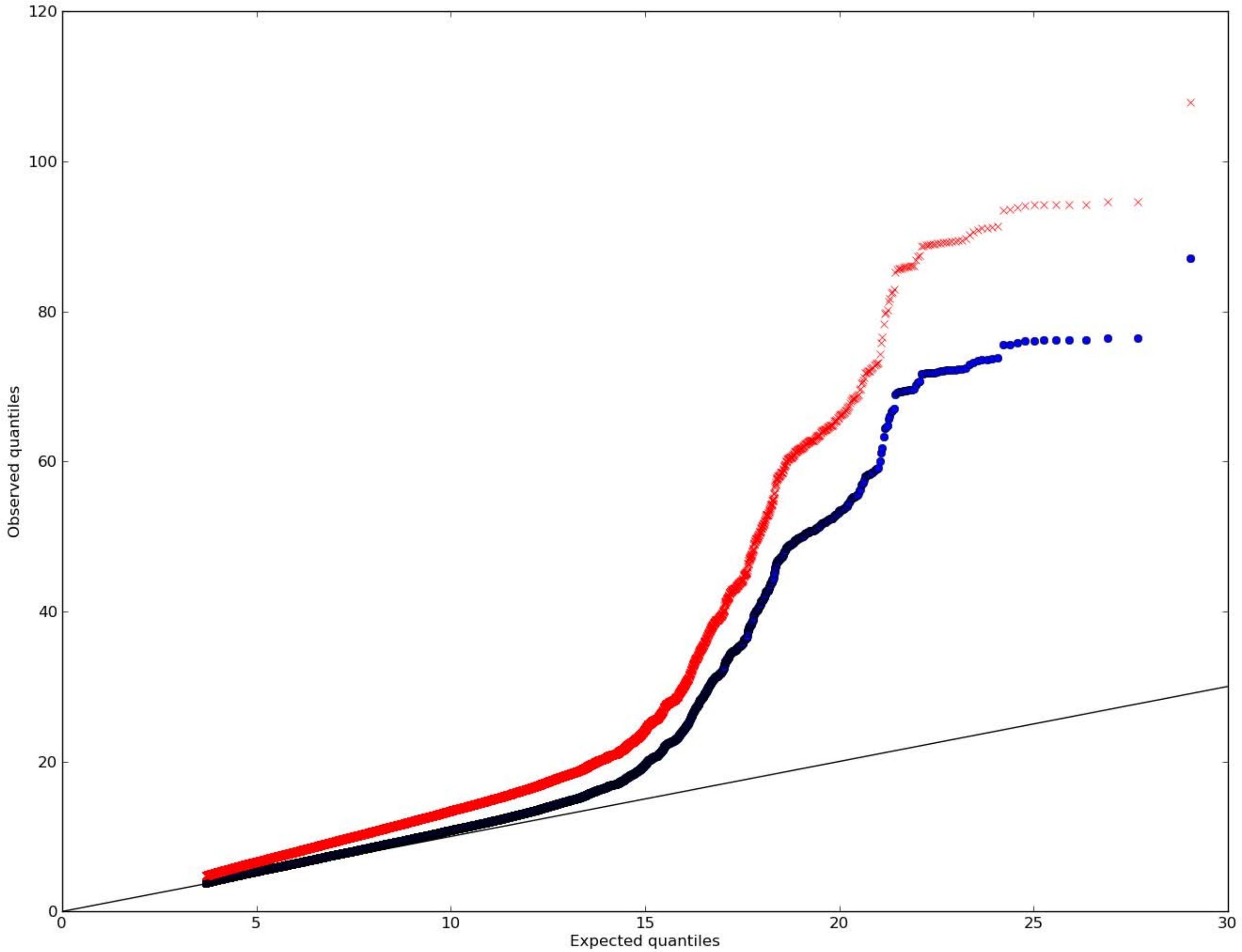

Supplementary Figure 1: Q-Q plot of chi-squared statistics from imputed variants tested for association with BCC in the Icelandic sample. Unadjusted values are shown in red, values adjusted using genomic control are shown in blue ($\lambda = 1.2374$). The expected (diagonal) values are indicated by a black line. Variants plotted have an information ≥ 0.8 and a minor allele frequency of $\geq 0.1\%$.

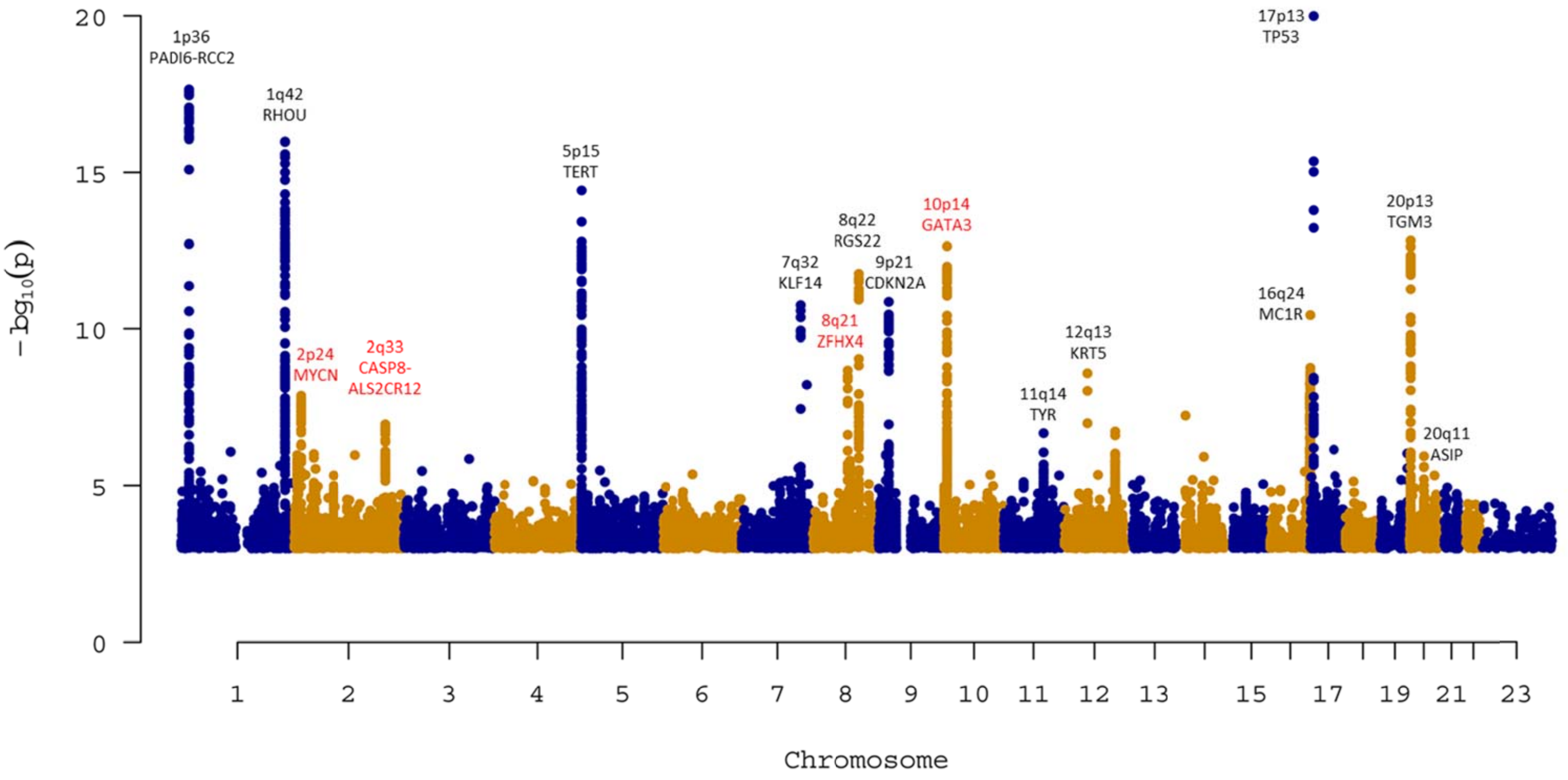

Supplementary Figure 2: Manhattan plot of association with BCC in the Icelandic sample, determined using logistic regression. Previously published loci are indicated in black, loci discussed in this article are indicated in red. Variants plotted have an information ≥ 0.8 and a minor allele frequency of ≥ 0.1%.

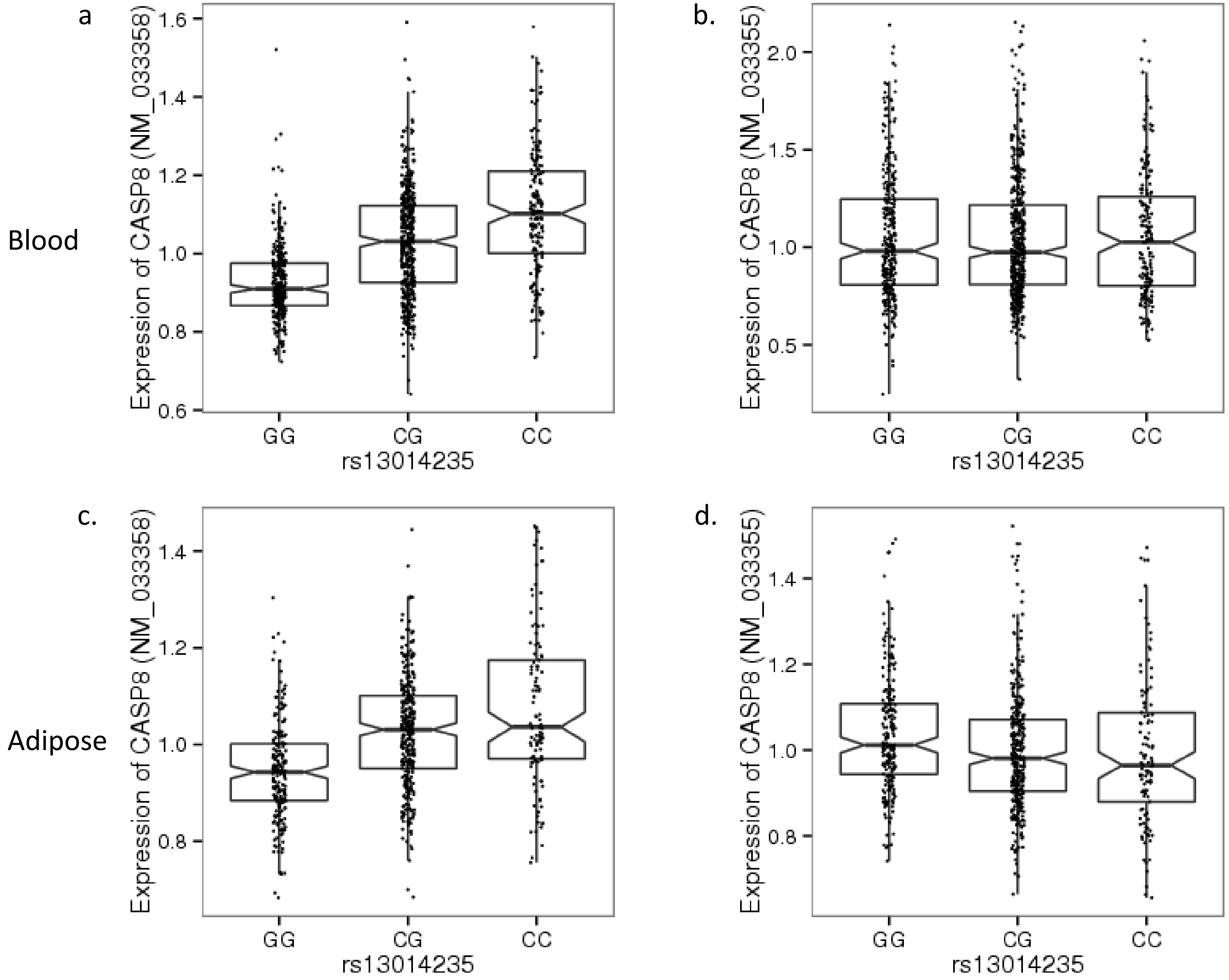
a
b.
c.
d.
Blood
Adipose
Expression of CASP8 (NM_033358)
Expression of CASP8 (NM_033355)
Expression of CASP8 (NM_033358)
Expression of CASP8 (NM_033355)
GG
CG
CC
rs13014235
rs13014235
rs13014235
rs13014235
0.6
0.8
1.0
1.2
1.4
1.6
0.5
1.0
1.5
2.0
0.8
1.0
1.2
1.4
0.8
1.0
1.2
1.4

Supplementary Figure 3: RNA expression microarray data for *CASP8* transcript variants, stratified by genotype of BCC risk variant rs13014235. RNA from blood and adipose tissue was hybridized to Agilent expression microarrays containing probes for the major *CASP8* transcript variants (NM_033355) and the variant encoding isoform E (NM_033358). Normalized expression levels were stratified by genotype of variant rs13014235, allele [C] being the risk allele for BCC. Effects and *P*-values were determined using multivariable linear regression. The box-plots show median, 25th and 75th percentiles (boxes) and 5th and 95th percentiles (vertical lines). Individual points for each sample are also plotted. (a) expression of the isoform E transcript variant NM_033358 in blood. eQTL result coded to rs13014235[C] is $\beta = 0.0384$, $P = 1.9 \times 10^{-39}$. (b) expression of the major transcript variants NM_033355 in blood. $\beta = 0.0065$, $P = 0.31$. (c) expression of the isoform E transcript variant NM_033358 in adipose tissue. $\beta = 0.0283$, $P = 3.1 \times 10^{-19}$. (d) expression of the major transcript variants NM_033355 in adipose tissue. $\beta = -0.0109$, $P = 0.0070$.

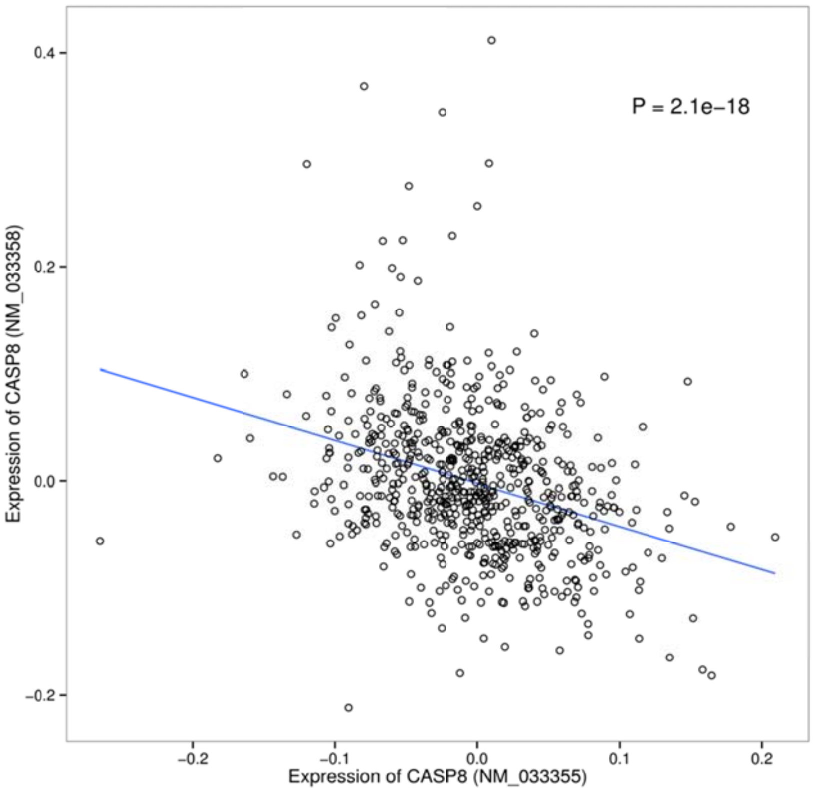

Supplementary Figure 4: Expression of *CASP8* major isoform transcript variants is inversely correlated with expression of the isoform E-encoding transcript variant. RNA from adipose tissue from 673 individuals was hybridised to Agilent expression microarrays. Log_{10} transformed expression levels (adjusted for age and gender) for the probe NM_033358 which detects the isoform E-encoding *CASP8* transcript variant were regressed on expression levels for the probe NM_033355 which detects the major *CASP8* isoform transcript variants.

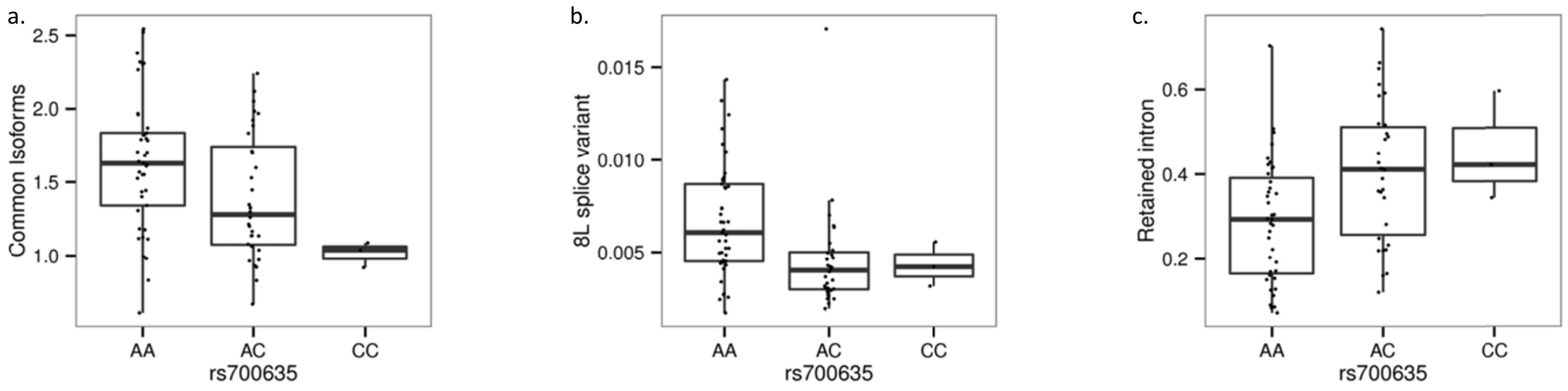

Supplementary Figure 5: RT-PCR of *CASP8* transcripts in 76 blood-derived RNA samples using primers as depicted in Figure 2. Normalized expression levels were stratified by genotype of variant rs700635, allele [C] being the risk allele for BCC. Effects and *P*-values were determined using multivariable linear regression. The box-plots show median, 25th and 75th percentiles (boxes) and 5th and 95th percentiles (vertical lines). Individual points for each sample are also plotted. (a) RT-PCR with primers covering the exon9-exon10 junction was used to detect the common *CASP8* isoform transcripts. eQTL β = -0.188, *P* = 0.0050. (b) RT-PCR with reverse primer in exon 8L unique sequence, forward primer in exon 6 was used to detect exon 8L-specific transcripts. eQTL β = -0.294, *P* = 0.0043. (c) RT-PCR using primers unique to the retained intron 8 sequence upstream of the NM_033358 probe sequence. eQTL β = 0.349, *P* = 0.0021.

a. Exon 8 ^ Exon 9 b. Exon 8L ^ Exon 9 c. Exon 8 ^ Probe Exon

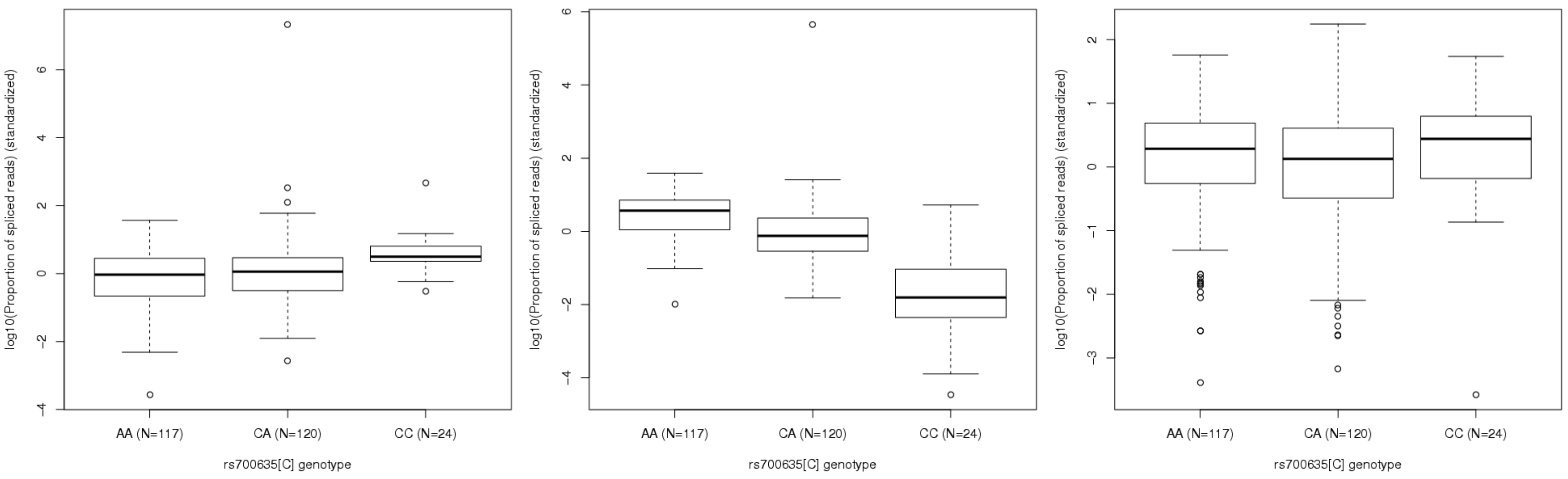

Supplementary Figure 6: Efficiency of utilization of *CASP8* exon 8 and exon 8L splice donors stratified by rs700635 genotype. (a) The number of RNA-seq reads crossing the exon 8 ^ exon 9 splice junction was expressed as a ratio relative to the median of read counts in exon 8 (chr2:201849794-201849936). Linear regression was carried out on log transformed ratios standardized so that the response variable had mean 0 and standard deviation 1. $\beta = 0.30$ s.d. units, $P = 0.0017$, coded to rs700635[C]. The box-plots show median, 25th and 75th percentiles (boxes) and 5th and 95th percentiles (ticks). Individual points are also plotted for outliers. (b) The number of RNA-seq reads crossing the exon 8L ^ exon 9 splice junction was expressed as a ratio relative to the median of read counts in sequences specific to exon 8L (chr2:201849937-201850072). Linear regression was carried out as in (a). $\beta = -0.85$ s.d. units, $P = 6.2 \times 10^{-22}$. (c) The number of RNA-seq reads crossing the exon8 ^ NM_033358 probe exon splice junction was expressed as a ratio relative to the median of read counts in exon 8. Linear regression was carried out as in (a). $\beta = 0.014$ s.d. units, $P = 0.89$.

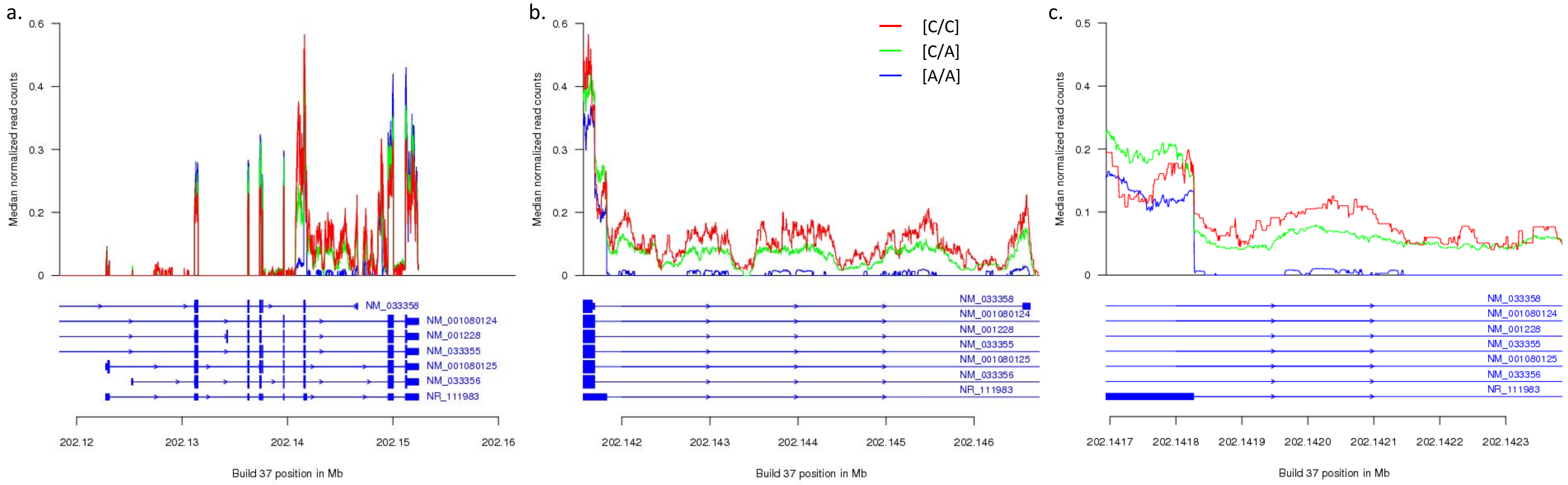

Supplementary Figure 7: Carriers of the rs700635[C] BCC risk allele show preferential retention of *CASP8* intron 8 in RNA from sun exposed skin. RNA-seq data obtained from sun exposed skin of genotyped donors was provided by the GTEx consortium. Donors were n=11 rs700635[C/C] homozygotes (shown in red), n = 58 rs700635[C/A] heterozygotes (shown in green) and n = 62 rs700635[A/A] homozygotes (shown in blue). The X-axis is the genomic position in Mb (hg19/Build 37). The Y-axis is, for each genotypic group, the median count of normalized reads (normalized for each individual to the total number of aligned reads). The structure of the RefSeq transcript variants is shown beneath the graphs. (a) shows the genomic region covering the coding exons of *CASP8*. (b) zoom showing the exon 8, exon 8L region extending through intron 8 to the minor exon targeted by the NM_033358 probe. (c) zoom showing the splice junction between exon 8L and intron 8.

Supplementary Table 1: Age at Diagnosis of Current and Previously Published BCC Associated Variants

						Current BCC Association Result[a]			Age at Diagnosis[b]	
SNP	Allele[c]	Allele Frequency	Chr	Position	Locus[d]	OR	P	Publication	β (years)	P
rs57244888	T	0.899	2	16,325,626	MYCN	1.30	$1.4x10^{-8}$	Current	-0.562	0.095
rs13014235	C	0.456	2	201,923,737	CASP8-ALS2CR12	1.15	$1.6x10^{-7}$	Current	-0.198	0.28
rs28727938	C	0.938	8	77,641,094	ZFHX4	1.43	$2.1x10^{-9}$	Current	-0.724	0.10
rs73635312	G	0.874	10	8,976,004	GATA3	1.36	$2.3x10^{-13}$	Current	-0.561	0.074
rs7538876	A	0.356	1	17,594,950	PADI6-RCC2	1.25	$7.6x10^{-17}$	14	-0.549	0.0031
rs801114	G	0.330	1	227,064,458	RHOU	1.23	$8.3x10^{-14}$	14	-0.357	0.068
rs401681	C	0.546	5	1,375,087	TERT	1.21	$8.6x10^{-13}$	17	-0.246	0.20
rs11170164	T	0.087	12	51,199,935	KRT5	1.29	$9.3x10^{-9}$	10	-0.451	0.13
rs2151280	G	0.532	9	22,024,719	CDKN2A/B	1.19	$8.7x10^{-11}$	10	-0.095	0.60
rs157935	T	0.675	7	130,236,093	KLF14	1.21	$4.2x10^{-11}$	10	-0.520	0.011
rs78378222	G	0.018	17	7,512,477	TP53	2.07	$1.0x10^{-20}$	15	-0.838	0.090
rs214782	G	0.171	20	2,229,970	TGM3	1.28	$2.3x10^{-13}$	16	0.006	0.97
rs7006527	A	0.858	8	101,093,681	RGS22	1.32	$2.4x10^{-12}$	16	0.265	0.35
rs1800407	T	0.050	15	25,903,913	OCA2	1.17	0.0051	11	0.687	0.086
rs12203592	T	0.119	6	341,321	IRF4	1.16	$1.8x10^{-4}$	12	0.261	0.35
rs12210050	T	0.159	6	420,489	EXOC2-IRF4	1.12	0.0011[e]	[f]	0.283	0.26
rs7335046	G	0.123	13	98,839,739	UBAC2	1.03	0.53	[f]	0.050	0.85

[a] association results obtained in the Icelandic population sample from the current analysis

[b] association with age at diagnosis determined by linear regression adjusted for gender and year of birth. Association is based on chip-typed individuals only.

[c] note that in this table the alleles are coded as the at-risk allele for BCC

[d] each locus is named by nearby gene(s). This does not imply that the named gene necessarily mediates the pathogenic effect of the variant.

[e] this association does not survive adjustment for the effect of rs12203592

[f] Nan H et al. Hum. Mol. Genet. **20**, 3718-3724 (2011).

Supplementary Table 2: Association between rs700635[C] and expression of selected regions of CASP8 based on RNA-seq data.

Region Name	Location	Tissue	β (SD units)[a]	P[a]
NM_033358 probe region	chr2:201854800-201854883	blood	0.94	$1.6x10^{-27}$
Exon 8	chr2:201849794-201849936	blood	-0.36	$1.4x10^{-4}$
Exon 8 & 8L	chr2:201849794-201850072	blood	-0.36	$1.3x10^{-4}$
Exon 8L only	chr2:201849937-201850072	blood	-0.63	$6.3x10^{-12}$
Intron 8	chr2:201850072-201854800	blood	0.96	$3.0x10^{-29}$
CASP8 whole gene	all RefSeq exons combined	blood	-0.65	$1.7x10^{-12}$
NM_033358 probe region	chr2:201854800-201854883	sun exposed skin	0.53	$7.1x10^{-5}$
Exon 8	chr2:201849794-201849936	sun exposed skin	0.31	0.022
Exon 8 & 8L	chr2:201849794-201850072	sun exposed skin	0.32	0.019
Exon 8L only	chr2:201849937-201850072	sun exposed skin	0.22	0.11
Intron 8	chr2:201850072-201854800	sun exposed skin	1.17	$1.8x10^{-24}$
CASP8 whole gene[b]	all RefSeq exons combined	sun exposed skin	-0.64	$7.2x10^{-9}$

[a] Effect and *P* value determined by regressing rs700635[C] allele count against normalized median read counts over the region indicated.

[b] This eQTL result was obtained directly from the GTEx portal (see URLs).
